# An Artificial Biomimetic Catalysis Converting CO_2_ to Green Fuels

**DOI:** 10.1186/s11671-017-2293-4

**Published:** 2017-09-11

**Authors:** Caihong Li, Zhiming Wang

**Affiliations:** The Institute of Fundamental and Frontier Science, University of Electronic Science and Technology of China, Chengdu, 610051 China

**Keywords:** Molecular iron catalyst, CO_2_, CO, CH_4_, Visible light

## Abstract

Researchers devote to design catalytic systems with higher activity, selectivity, and stability ideally based on cheap and earth-abundant elements to reduce CO_2_ to value-added hydrocarbon fuels under mild conditions driven by visible light. This may offer profound inspirations on that. A bi-functional molecular iron catalyst designed could not only catalyze two-electron reduction from CO_2_ to CO but also further convert CO to CH_4_ with a high selectivity of 82% stably over several days.

## Background

Social development and the energy crisis have increased the demand on chemical fuels. Furthermore, the increasing concentrations of CO_2_ in the atmosphere owing to human activities such as excessive combustion of fossil fuel, exhaust gas emission and respiration have had a series of terrible impacts including global warming, desertification, and sea level rise. One of the greatest innovations in mitigation of energy crisis and greenhouse effect was converting greenhouse gases CO_2_ into fuel chemical feedstock compounds such as CH_4_, CO, and other small molecules with visible light (it is called photoreduction, in scientific jargon) [1]. The most remarkable superiority of photoreduction is that it can be driven by visible light compared to electroreduction which is activated by applied voltage or thermal reduction with high temperature. In addition, approximately half of solar light is located in the visible range. However, the low production rate and selectivity because of multiple reaction pathways and a variety of products severely limit large-scale practical application of CO_2_ reduction.

The challenges in catalytic reduction of CO_2_ to value-added fuels ideally based on cheap and earth-abundant elements rather than on precious metals are efficiency, stability, and selectivity [2]. So far, the main methods addressing these challenges have fallen into three categories: screening transition metals [3] with high catalytic activity as active sites such as Fe, Co, and Ni; formation of organic macrocyclic structures to enhance long-term stability [4]; and ligand modification [5] to strengthen the desired product selectivity. In each approach, the selected metal element and the structural design both contribute to the final catalytic performance and product selectivity.

Organic macrocyclic structures (OMS) chaining transition metal elements are very popular catalysts used in CO_2_ reduction, where the metal elements act as catalytic active sites to adsorb and bind the CO_2_ molecules [6]. Microporous OMS can offer larger specific surface area, i.e., more active sites to support catalytic reactions. Nevertheless, the original OMS may not possess the optimized catalytic performance. Structure optimization such as ligand modification would improve the catalytic activity especially product selectivity by inducing internal interactions like H bonds which can stabilize the specific intermediates in favor of gaining desired products.

## Experimental

Inspired from the photosynthesis of plants, Rao et al. [7] creatively designed a biomimetic photocatalytic system based on a molecular iron catalyst which miraculously produced CH_4_ from CO_2_ at ambient temperature and pressure. Such a frontier and significant discovery was published in Nature.

Rao and co-workers judiciously designed an iron (transition metal element) tetraphenylporphyrin (organic macrocyclic structure) complex functionalized with trimethylammonio groups (ligand modification) as the catalyst to reduce CO_2_. This catalytic system was operated in a CO_2_-saturated acetonitrile (CH_3_CN) solution containing a visible-light photosensitizer aiming to capture photons from light irradiation and afford energy (hυ) for the redox reactions as well as a sacrificial electron donor used to provide electrons on photo-induced command by photosensitizer to reduce CO_2_. The whole system was significantly stable and driven by visible light (*λ* > 420 nm) at 1 atm and room temperature.

## Discussion

Furthermore, Rao et al. first reported that the above catalytic system whose catalyst was known as the most efficient and selective molecular electro-catalyst for reducing CO_2_ to CO in two-electron process, could also be applied for the eight-electron reduction [8] from CO_2_ to CH_4_. They found an entirely new function of this molecular iron catalyst under moderate conditions. Meanwhile the authors analyzed and verified the reaction mechanism of two-step procedure that first reduced CO_2_ to CO and then converted CO to CH_4_ with 82% of the CH_4_ selectivity by isotope labeling experiments and blank experiments for the first time. Besides, they also found that a meta-acid condition could play a role of proton donor as well as H bond donor towards stabilized intermediates [7, 9] but unwished by-product hydrogen selectivity would increase either.

**Fig. 1:**
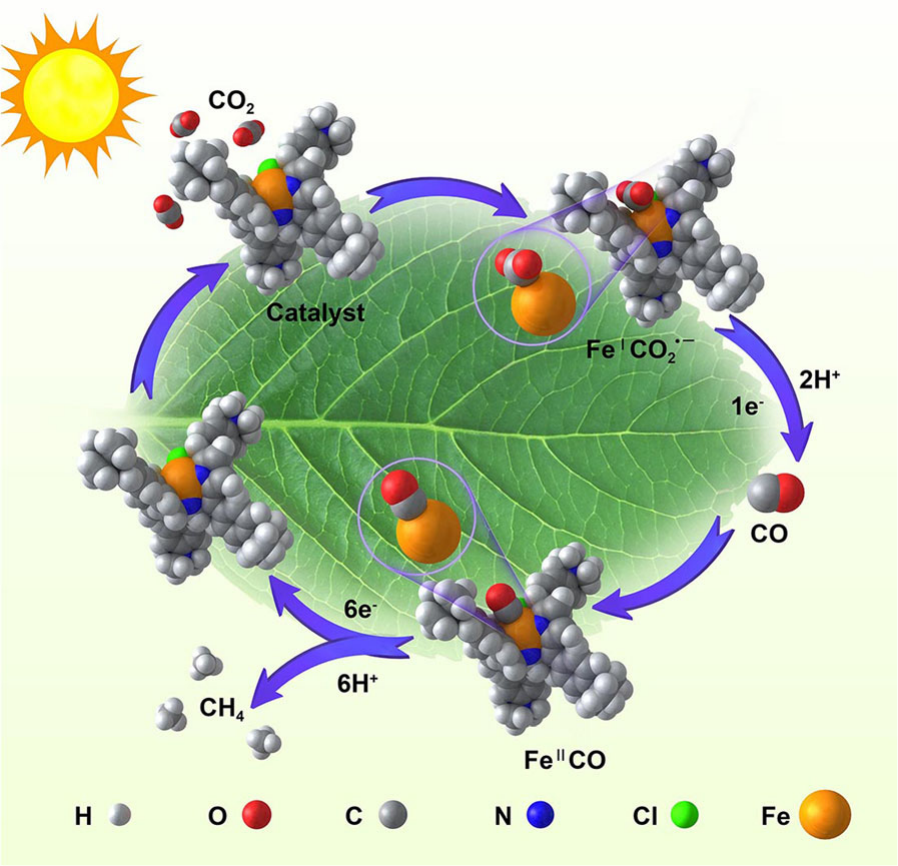
A sketch map of photoreduction from CO_2_ to CH_4_

Greenhouse gas CO_2_ molecules were adsorbed on the surface of catalyst or more precisely on the metal Fe active sites and distorted from the linear structure to a certain angle; thus CO_2_ molecules were activated [10] and formed Fe–CO_2_ adduct. In addition, this adduct was further protonated reacting with H^+^ from solution and formed Fe–CO adduct dehydrating a H_2_O molecule. The intermediates of CO could be obtained through hydrogenation at this time. Then, CO molecules were bound to the metal active sites again through subsequent multistep protonation and electron transfer process and proceeded to yield CH_4_ gas eventually desorbing from the catalyst surface. Later, this catalyst reused to next catalytic cycle of CO_2_ molecules (Fig. 1).

## Conclusions

The catalytic system they designed was bi-functional, for catalyzing not only relatively simple two-electron reduction to CO but also eight-electron reduction to CH_4_ utilizing only one catalyst at very easily satisfied conditions. This was a profound progress because a catalyst can catalyze efficiently a certain reaction generally. The uplifting discovery of Rao et al. aroused great interest on photoreduction of CO_2_ to value-added CH_4_ and has inspired future efforts in this field. A drawback of this report is that the authors have not deciphered the reduction mechanism in greater detail yet; otherwise, it will help to develop more efficient catalytic system improved from mechanism aspects. Cheaper gas fuel may be produced when the productive rate is improved via optimization of structure and conditions.

The catalytic system designed by Rao et al. has other promising properties besides these described here. For example, it can convert toxic gas CO to green fuel CH_4_ just by light irradiation. Such a simple but significant conversion might guide a new craze to turn waste into wealth environmentally and efficiently. The application and development of their discovery might form the basis of a new branch of CO_2_ photoreduction or toxic gas conversion.
